# Homœopathy Fairly Represented

**Published:** 1855-06

**Authors:** Wm. Henderson


ART. V.—*Homœopathy Fairly Represented.* By WM. HENDERSON, M. D.

This is the second Edinburgh edition of a book since republished on this side. The American reprint has had its notice at our hands, and it is only necessary to say of this that, like most Edinburgh publications, its typography is very neat.

We are a little surprised, however, that so ill-tempered an essay as this should have reached a second edition. The explanation is probably to be found in the fact that the uncultivated and ignorant lay-reader is as fully able to appreciate the logic of the thing, as the most erudite medical scholar. It is pure doctrine. Experiment, fact, reason, common-sense, all go for nothing in such an argument; volubility wins the day, and the man who knows nothing of anatomy, physiology, chemistry, medicine, or medical logic, is delighted to find himself addressed upon the level of his own understanding. The fool becomes a wise man, and thenceforth his talk is of dilutions, potencies, and infinitesimals, and he goes gabbling about his social circle, verifying the sententious axiom that "it is somewhat difficult, if not actually impossible, for a man to speak well upon a subject whereof he knows nothing."

